# Humidity Sensing in Graphene-Trenched Silicon Junctions via Schottky Barrier Modulation

**DOI:** 10.3390/nano15130985

**Published:** 2025-06-25

**Authors:** Akeel Qadir, Munir Ali, Afshan Khaliq, Shahid Karim, Umar Farooq, Hongsheng Xu, Yiting Yu

**Affiliations:** 1 School of Information Engineering, Xi’an Eurasia University, Xi’an 710065, China; akeelqadir@eurasia.edu (A.Q.); shahid@nwpu.edu.cn (S.K.); 2 Research Center of Smart Sensing Chips, Ningbo Institute of Northwestern Polytechnical University, Ningbo 315103, China; 3 Key Laboratory of Micro/Nano Systems for Aerospace (Ministry of Education), Shaanxi Province Key Laboratory of Micro and Nano Electro-Mechanical Systems, Northwestern Polytechnical University, Xi’an 710072, China; 4 Laboratory of Single-Photon Detection and Imaging Techniques, Zhejiang Engineering Research Center for Edge Intelligence Technology and Equipment, School of Information and Electrical Engineering, Zhejiang University City College, Hangzhou 310015, China; munir_ali@zju.edu.cn; 5 College of Physics and Electronic Information Engineering, Zhejiang Normal University, Jinhua 321004, China; afshan.k@zju.edu.cn; 6 Department of Mechanical Engineering, University of Colorado Boulder, Boulder, CO 80309, USA; engr.umarfarooq@gmail.com; 7 Industry-Education-Research Institute of Advanced Materials and Technology for Integrated Circuits, Anhui 11 University, Hefei 230601, China; xhs@ahu.edu.cn; 8 Key Laboratory of Scale Manufacturing Technologies for High-Performance MEMS Chips of Zhejiang Province, Key Laboratory of Optical Microsystems and Application Technologies of Ningbo City, Ningbo Institute of Northwestern Polytechnical University, 218 Qingyi Road, Ningbo 315103, China

**Keywords:** graphene-trenched silicon Schottky junction, sensitivity, humidity sensor, relative humidity

## Abstract

In this study, we develop a graphene-trenched silicon Schottky junction for humidity sensing. This novel structure comprises suspended graphene bridging etched trenches on a silicon substrate, creating both free-standing and substrate-contacting regions of graphene that enhance water adsorption sensing. Suspended graphene is intrinsically insensitive to water adsorption, making it difficult for adsorbed H_2_O to effectively dope the graphene. In contrast, when graphene is supported on the silicon substrate, water molecules can effectively dope the graphene by modifying the silicon’s impurity bands and their hybridization with graphene. This humidity-induced doping leads to a significant modulation of the Schottky barrier at the graphene–silicon interface, which serves as the core sensing mechanism. We investigate the current–voltage (I–V) characteristics of these devices as a function of trench width and relative humidity. Our analysis shows that humidity influences key device parameters, including the Schottky barrier height, ideality factor, series resistance, and normalized sensitivity. Specifically, larger trench widths reduce the graphene density of states, an effect that is accounted for in our analysis of these parameters. The sensor operates under both forward and reverse bias, enabling tunable sensitivity, high selectivity, and low power consumption. These features make it promising for applications in industrial and home safety, environmental monitoring, and process control.

## 1. Introduction

Environmental awareness involves the sensing of multiple variables such as gases/vapors, light, temperature, and more. These sensors have been extensively applied in various sectors, including industrial and household security, environmental monitoring, process control, homeland security, e-agriculture, etc. [1,2,3,4,5,6]. Advancements in modern technologies like smart grids, smart homes, intelligent vehicles, and smart cities underscores the need for a new generation of multivariate environmental sensors. These sensors are designed to incorporate sensing elements with multi-response mechanisms capable of detecting and responding to various environmental impulses.

Graphene’s distinctive electronic band structure, excellent electrical conductivity, high carrier mobility, and the ability to tune its Fermi level through electrical, mechanical, or chemical techniques make it highly suitable for sensing applications [7,8]. Various device structures, such as Schottky junctions, field-effect transistors [9,10,11,12,13,14], and chemi-resistors [15,16], have been implemented to leverage these properties. Graphene–silicon Schottky junctions provide a unique platform for studying and manipulating graphene’s electronic properties. Unlike chemi-resistive platforms, where changes in graphene resistance are governed by Ohm’s law [17], the current rectification at graphene–silicon junction interfaces arises from the formation of a Schottky barrier. This barrier forms due to the built-in potential that impedes charge transport from graphene to silicon [18,19], since graphene’s Fermi level, which ranges between 4.6 and 4.7 eV, resides within silicon’s band gap.

Both n-type and p-type silicon substrates can be implemented in Schottky junction formation with graphene. p-type silicon has been reported to form a larger Schottky barrier height ${(\phi }_{b})$ of (0.61–0.78 eV) when compared to n-type silicon (0.52–0.73 eV) [20,21]. Upon the exposure of graphene to electron-acceptor molecules, the $\phi_{b}$ of the graphene–silicon junction device increases (decreases) for n-Si (p-Si) substrates. Correspondingly, the series resistance $\left(R_{s}\right)$ of the device drops (rises) for n-Si (p-Si) upon exposure to electron acceptors [22]. The incorporation of few-layer exfoliated graphene, bilayer graphene, and monolayer graphene onto n- and p-type silicon substrates [23,24,25,26], MoS_2_ [27], CdS [28], GaAs and GaN [29], and other 2D materials extended the application of graphene-semiconductor-based Schottky junctions to several areas, including photovoltaics, photodetectors, and chemical sensors [30,31,32,33,34]. So far, graphene–silicon Schottky junctions have been identified for their potential applications in gas sensing. These demonstrate high sensitivity for the detection of various gaseous species such as NH_3_, H_2_S, or H_2_ [35]. However, despite the great inherent sensing potential of this platform, very few attempts have been made to control their relative humidity (RH) sensing through silicon surface engineering (trench formation).

In order to regulate the humidity sensing of a graphene–silicon junction, we devised a novel graphene-trenched silicon Schottky diode. Trenches enable a platform featuring a dual-natured graphene layer. Both sensitive (graphene forming a Schottky junction with silicon) and insensitive (suspended graphene) regions are combined in one device. Graphene is not investigated as a channel, but rather as a material knob for tuning the device’s sensing plausibility. The RH is reflected through $\phi_{b}$, ideality factor (η), and $R_{s}$ probes. Their variations are investigated in relation to the area ratio of the trenched and non-trenched sections in the silicon window. Our humidity sensor, fabricated using cost-effective silicon technology, provides reliable humidity sensing and is readily scalable to array-level integration, offering a practical alternative to contemporary sensors that rely on advanced materials and complex fabrication techniques [36,37].

The remainder of this paper is organized as follows: Section 2 provides fabrication details for the graphene-trenched silicon Schottky junction. Section 3 comprises the measurement scheme. Section 4 provides a thoughtful discussion of the physical mechanisms affecting the extracted results. This is followed by Section 4, starting with Section 4.1, which discusses the current–voltage (I−V) characteristics under linear sweep voltage and RH increasing from fully dry (0.1%) to highly saturated (90%) humidity conditions. Section 4.2 discusses the extraction of tunable figures of merit e.g., $\phi_{b},\eta$, and $R_{s}$, and normalized sensitivity ($S_{T}$). This subsection ends with a discussion of various plausible futuristic research directions. Finally, in Section 5, we conclude this work.

## 2. Experimental Section

### 2.1. Device Fabrication

**Figure 1:**
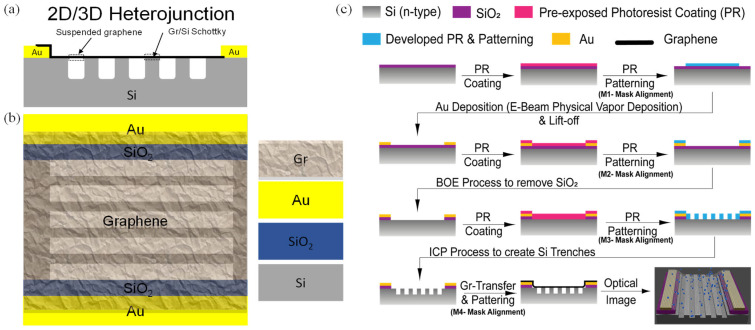
(**a**) The cross-section and (**b**) top-view of a graphene-trenched silicon Schottky junction are shown schematically. Graphene is laying freely suspended and in physical-contact configurations with silicon. (**c**) A step-by-step work flow chart of the involved fabrication processes is displayed.

Figure 1a shows a cross-section and Figure 1b displays a top-view schematic image of the graphene-trenched silicon Schottky junction. Graphene is laid in both suspended and physical-contact configurations with the silicon substrate. A step-by-step fabrication work flow chart is shown in Figure 1c. The fabrication process starts with the deployment of an n-type silicon substrate of resistivity 1–10 Ω×cm having a pre-deposited and polished 300 nm thick ${SiO}_{2}$ layer onto it. Firstly, to deposit metallic electrodes onto the device, the substrate is spin-coated with 5350 photoresist (PR), followed by ultraviolet (UV) photolithography. After the subsequent development, the deposition of Au/Ti (60 nm/5 nm) metals is executed, which is then followed by the lift-off process. After a subsequent UV photolithography process, the sample is placed into buffered oxide etchant to expose a silicon window with an area of 500 µm×500 µm. A third PR spin-coating step, followed by successive UV photolithography, was performed to enable the silicon window area for the Inductively Coupled Plasma-Reactive Ion Etching process to dig trenches of various thicknesses $\left(3,5,7,9\right)\;µm$ and fix depth of 3 µm, as shown in Figures S2–S5. Subsequently, monolayer graphene, grown via chemical vapor deposition (CVD) on a copper foil (purchased from Zhongke Crystal Materials, Dongguan, China), was first coated with polymethyl methacrylate (PMMA, ALLRESIST AR-26) using spin-coating at 5000 rpm for 60 s. The copper foil was then etched using a solution of CuSO_4_, HCl, and H_2_O (10 g:50 mL:50 mL) for 5 h, followed by rinsing in deionized water. The PMMA-supported graphene was transferred onto the prepared silicon substrate to conformally cover both trenched and untrenched regions in the silicon window and metal electrodes. PMMA was then removed with acetone, and the surface was cleaned using isopropyl alcohol (IPA). Excess graphene was eliminated via O_2_ plasma after patterning. Finally, eutectic GaIn was applied to the backside of the silicon substrate to form an Ohmic contact, serving as the back electrode.

### 2.2. Raman Spectroscopy and Measurement Setup

**Figure 2:**
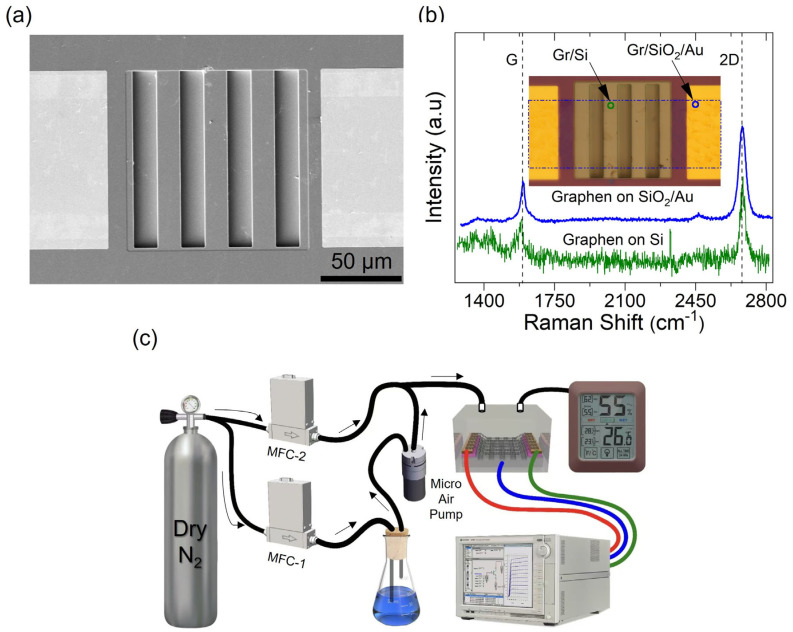
(**a**) A scanning electron microscopy (SEM) image of the fabricated graphene–trenched silicon Schottky diode is presented. The related scale bar of 50 µm is also shown. (**b**) The RAMAN spectroscopy performed at the $Au/{SiO}_{2}$ and the Si sites are shown, revealing the characteristic peaks obtained for the transferred CVD-grown monolayer graphene. The inset shows an actual high-resolution micrograph of the device. The blue and red circles are shown at the exact locations where RAMAN spectroscopies are executed. (**c**) The custom-built measurement system, including the humidity controlling chamber and electrical inputs/outputs connected to the Agilent Semiconductor Analyzer B1500, is shown.

Figure 2a displays the scanning electron microscope (SEM) image of a graphene-trenched silicon Schottky junction. The related scale bar of 50 µm is also shown. The SEM image confirms the presence of parametrically fabricated trenches where graphene is in suspended morphology, while sharp and smooth physical contact corresponds to regions actually acting as graphene-silicon Schottky contact. Raman spectroscopy is implemented to both verify the presence and quality of a monolayer CVD-grown graphene sheet at $Au/{SiO}_{2}$ site, represented by the blue circle, and at the Si site emphasizing Schottky contact, signified by the green circle, as shown in the inset of Figure 2b.

The equipment employed for such spectroscopy is a RENISHAW RM2000 coupled with a 532 nm light. The spectroscopy confirmed the presence of graphene at both sites, while their typical characteristic peaks are shown over similar scales in Figure 2b. Two main peaks occur at $\sim 1589\;{cm}^{-1}$ (G peak) and $\sim 2700\;{cm}^{-1}$ (2D peak), signifying pristine graphene. They correspond to the E_2g_ phonon at the Brillouin zone and the overtone of the defect-activated D peak, respectively [38]. The D peak is due to the breathing modes of six-atom rings requiring a defect for their activation. It comes from TO phonons around the K point and is activated by intervalley double-resonance [39]. The $I_{2D}/I_{G}$ ratio is a commonly used metric to estimate the number of graphene layers: values ≥ 2 indicate monolayer graphene, between 1 and 2 suggest bilayer graphene, and <1 correspond to multilayer graphene [40,41]. Notably, bilayer graphene can exhibit twist-angle-dependent shifts and a broadening of the G and 2D Raman peaks, occasionally resulting in $I_{2D}/I_{G}$ ≥ 2 [42,43,44]. In our analysis, the sharp and well-defined G and 2D peaks with $I_{2D}/I_{G}$ ratios of 2.4 and 2 at the blue- and green-circled sites, respectively, confirm the presence of monolayer graphene. A defect-related D peak at ~$1320\;{cm}^{-1}$ informs us of the crystalline quality of the graphene. A very small $I_{D}/I_{G}$ ratio, achieved for the $Au/{SiO}_{2}$ site, indicates the high quality of the transferred graphene [40]. The same ratio is slightly compromised at the green-colored site, emphasizing the micro-nano ridges formed on the silicon’s surface due to a non-ideal ${SiO}_{2}$ etching process. Moreover, excessive noise results from thermal variations, laser fluctuations, and the relatively compromised graphene–silicon interface.

A complete measurement setup is illustrated in Figure 2c, designed to mimic a controlled relative humidity (RH) environment. A compact testing kit was developed for parallel characterization, featuring a chamber designed to accommodate a 2 cm × 2 cm silicon chip containing multiple devices with trench widths of 3 µm, 5 µm, 7 µm, and 9 µm, as illustrated in Supplementary Figure S1. An integrated device selector facilitates the targeted measurements of individual sensors without disrupting the ambient environment, enabling simultaneous and efficient data acquisition across the entire chip. In this study, an Agilent B1500 Semiconductor Device Analyzer was employed to record the current–voltage (I–V) characteristics under varying RH conditions.

## 3. Physical Mechanisms at Play

**Figure 3:**
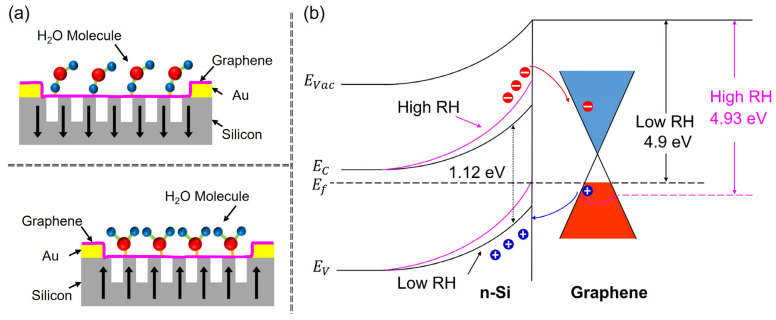
(**a**) The process of doping the sensitive graphene layer that is laid onto the semiconductor substrate is shown schematically. Graphene is p-doped when hydrogen atoms align close to it, acting as electron-withdrawing entities. It is n-doped when oxygen atoms are aligned closer to the graphene sheet, acting as electron-donating objects. (**b**) The humidity-sensing capability of our graphene-trenched silicon Schottky junction is displayed with a band diagram. Due to the presence of water vapors in the vicinity of the graphene-sensing layer, the shift in the substrate’s impurity bands is shown. The related hybridization changes with the graphene bands are also shown.

The charge transfers among various adsorbates and graphene are intrinsically dependent on the alignment and orientation of the adsorbed molecules [45]. The p-type doping of graphene is realized by adding atoms with fewer valence electrons than carbon, while the n-type doping is obtained by incorporating atoms with more valence electrons compared to carbon [46]. Various studies report the electron-withdrawing/electron-donating capabilities of multiple adsorbates implemented as p- or n-doping strategies in a wide range of 2D sensing materials [47,48,49,50]. Figure 3a emphasizes that the effect of p-doping graphene by $H_{2}O$ adsorption molecules will necessitate the alignment of hydrogen atoms toward graphene. Contrary to that, the effect of n-doping $H_{2}O$ adsorption onto graphene requires the alignment of oxygen atoms toward the graphene layer.

In our custom-built two-in-one sensor configuration, the freely suspended graphene is actually an insensitive section introduced to implement control. For both low and high concentrations of water molecules in close proximity to the suspended graphene, charge transfers between a graphene sheet and water molecules, resultantly manipulating the carrier density of graphene is negligible [51,52], whereas, at low concentrations of water molecules, the electronic properties of graphene, which is in physical contact with silicon, changes accordingly. The water molecules are capable of shifting semiconductor impurity bands and changing related hybridization with the graphene bands [53], while, at large RH, the dipole moments of the different molecules cumulate instead of balancing out, resulting in a total dipole moment capable of modifying the electronic properties of graphene, as shown in Figure 3b.

**Figure 4:**
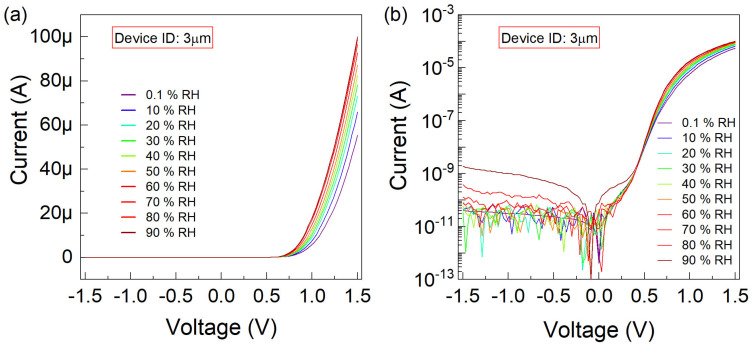
The current–voltage (I−V) characteristics are plotted, for the graphene-trenched silicon Schottky junction with a trench width of 3 µm, (**a**) linearly and (**b**) logarithmically versus the sweeping voltage (V) varying between −1.5 –1.5 V. The RH conditions of $\left(0.1,10,20,30,40,50,60,70,80,90\right)\;\%$ are parametrically implemented.

**Figure 5:**
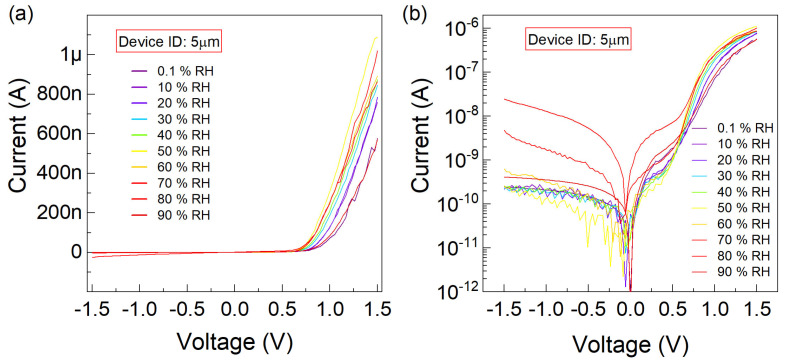
The current–voltage (I−V) characteristics are plotted, for the graphene-trenched silicon Schottky junction with a trench width of 5 µm, (**a**) linearly and (**b**) logarithmically versus the sweeping voltage (V) varying between −1.5 –1.5 V. The RH conditions of $\left(0.1,10,20,30,40,50,60,70,80,90\right)\;\%$ are parametrically implemented.

**Figure 6:**
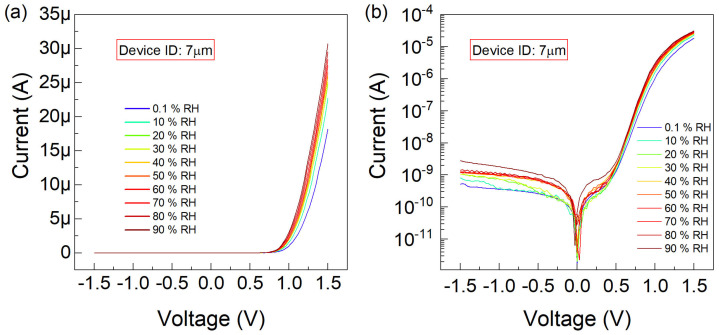
The current–voltage (I−V) characteristics are plotted, for the graphene-trenched silicon Schottky junction with a trench width of 7 µm, (**a**) linearly and (**b**) logarithmically versus the sweeping voltage (V) varying between −1.5 –1.5 V. The RH conditions of $\left(0.1,10,20,30,40,50,60,70,80,90\right)\;\%$ are parametrically implemented.

**Figure 7:**
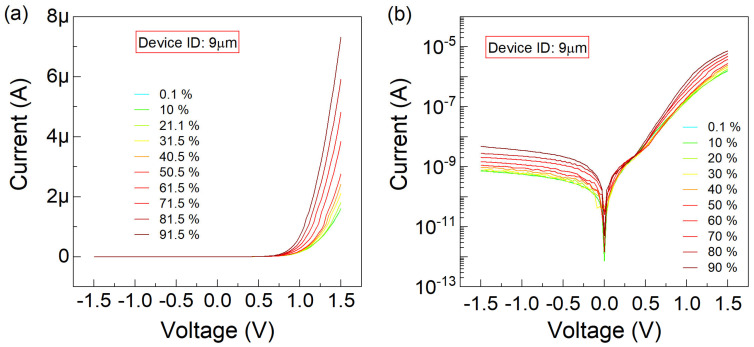
The current–voltage (I−V) characteristics are plotted, for the graphene-trenched silicon Schottky junction with a trench width of 9 µm, (**a**) linearly and (**b**) logarithmically versus the sweeping voltage (V) varying between −1.5 –1.5 V. The RH conditions of $\left(0.1,10,20,30,40,50,60,70,80,90\right)\;\%$ are parametrically implemented.

The room temperature work functions of graphene and n-type silicon are 4.9 and 4.05 eV, respectively. When these two materials are joined together using the techniques and processes expressed in Figure 1c, a Schottky junction is expected. The I−V curves of the devices presented in Figure 4, Figure 5, Figure 6 and Figure 7 are indeed characteristics of a Schottky diode [29]. We derived the following I–V characteristics of a Schottky diode utilizing Landauer transport formalism with the Crowell–Sze method for thermionic emission and diffusion of carriers over a barrier:

(1) $$I=\left[\frac{eD_{o}}{A.\tau_{i}}{\left(k_{B}T\right)}^{2}\left(\frac{\phi_{b}}{k_{B}T}+1\right)\right]exp\left(-\sqrt{\chi \delta }-\frac{{e\phi }_{b}}{k_{B}T}\right)\left[exp\left(\frac{e(V-IR_{s})}{{\eta k}_{B}T}\right)-1\right]$$

where $k_{B}$ is the Boltzmann constant, T is the absolute temperature, e is the elementary charge, $D_{o}$ = $2/\pi {\left(\hbar v_{F}\right)}^{2}$ is the density of states of graphene, χ is the average barrier height, δ is the oxide thickness, $v_{F}$ is the Fermi velocity in graphene, and ħ is the reduced Planck’s constant. The ${\tau_{i}}^{-1}$ represents the injection rate of carriers from silicon to graphene and is related to the silicon–graphene and metal–graphene coupling energy. Equation (1) is linear for V ≫ *η*$k_{B}$*T* until a larger bias voltage (approximately 1 *V*) where $R_{s}$ becomes prevalent. The slope of the linear segment of the I−V plot determines η, while the intercept on the y-axis gives the saturation current, which can be used to calculate $\phi_{b}$ [54].

## 4. Results and Discussions

### 4.1. Current vs. Voltage Characteristics

Through the formation of trenches inside the silicon window, the actual area of the graphene sheet involved in vapor sensing is reduced. The I−V signatures extracted from all four devices having trench widths of $\left(3,\;5,\;7,\;9\right)\;µm$ exhibit a reduction in the density of the states, which can be subsequently manipulated through the presence of bipolar water molecules. This is justified as hanging graphene acts insensitive. The involved areas are related to each other through

(2) $$A_{GS}=A_{HG}+A_{G-Si}$$

Here, $A_{GS}$ is the area of the patterned square shaped graphene, $A_{HS}$ corresponds to area of freely suspended graphene, while $A_{G-Si}$ is the area of actual graphene-silicon junction. The vapor sensing capability of graphene-trenched silicon Schottky diode is approximately linearly related to $A_{G-Si}$. According to the thermionic emission I−V transportation model presented in Equation (1), as humidity increases, the forward current rises due to reduction in junction resistance caused by the adsorption of water molecules and the subsequent charge transfer process.

Figure 4a displays the linear scale I−V curves for the graphene-trenched silicon Schottky junction characterized with $W_{T}$ of 3 µm, while the corresponding logarithmic I-linear V data is presented in Figure 4b. The gate voltage is swept from −1.5 V to +1.5 V. In this case, the difference between the minimum and maximum currents (I) at a forward bias of 1.5 V obtained at RH levels of 0.1% and 90% is 43 µA.

Similarly, for the device characterized with a $W_{T}$ of 5 µm, under comparable RH conditions and a sweeping gate bias V, the analogous data to that presented in Figure 4 is shown below in Figure 5. The difference between the minimum and maximum currents (I) at a forward bias of 1.5 V obtained at RH levels of 0.1% and 90% is 0.47 µA.

Next, the graphene-trenched silicon Schottky junction characterized with $W_{T}$ of 7 µm was tested under identical RH and electrical biasing conditions. The equivalent data compared to that shown in Figure 4 and Figure 5 is displayed in Figure 6. In this case, at a forward bias of 1.5 V under RH levels of 0.1% and 90%, the difference between the minimum and maximum currents occurs as 15 µA.

Finally, the graphene-trenched silicon Schottky junction characterized with $W_{T}$ of 9 µm was investigated under the same RH conditions with a sweeping gate bias (V). Figure 7 presents analogous data compared to that provided in Figure 4, Figure 5 and Figure 6. At a forward bias of 1.5 V and RH levels of 0.1% and 90%, the difference between the minimum and maximum current is 5.6 µA.

The s-shaped kinks observed in the forward biased measurement conditions observed via logarithmic I signatures plotted previously are caused by the presence of very thin ${SiO}_{2}$ layers. These layers may either not be fully removed during fabrication or form naturally due to exposure to ambient air.

### 4.2. Tunable Figures of Merit

**Figure 8:**
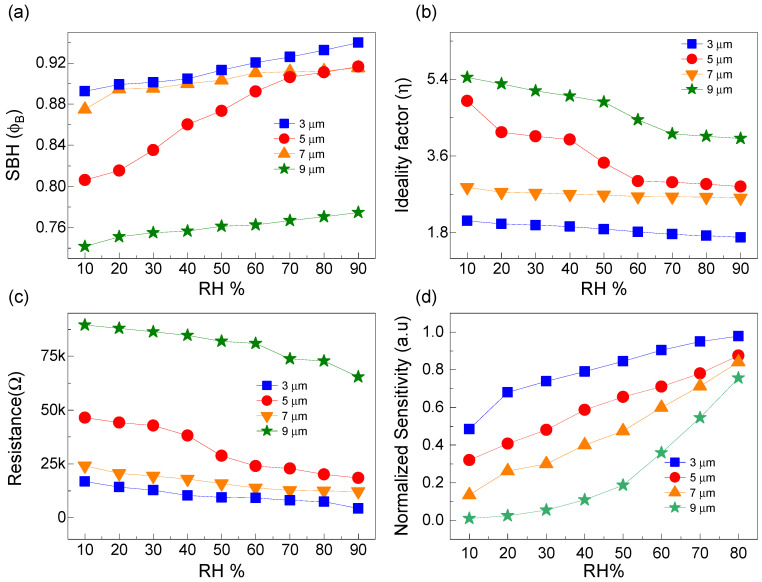
For the employed graphene-trenched silicon Schottky junctions characterized with $W_{T}$ of 3, 5, 7, and 9 µm tested under various humidity conditions, different evaluated figures of merits are plotted simultaneously as (**a**) Schottky barrier height ${(\phi }_{b})$, (**b**) ideality factor (η), (**c**) series resistance ${(R}_{s}),$ and (**d**) normalized sensitivity ${(S}_{T}).$.

The $\phi_{b}$ is essentially a figure of merit employed to estimate the ease or difficulty carriers experience when transporting across the graphene–silicon junction. The reverse-biased current is influenced not only by $\phi_{b}$, but also by the carrier injection ratio and the coupling energy of the interface contact. The $\phi_{b}$ can be adjusted by adsorbed molecules via graphene. We plot the $\phi_{b}$ values of the four graphene-trenched silicon Schottky junctions against RH levels of 10%, 20%, 30%, 40%, 50%, 60%, 70%, 80%, and 90% in Figure 8a. From the $\phi_{b}$–RH curves, a noticeable linear increase is observed. For a trench width of 3 µm, $\phi_{b}$ increases from 0.895 to 0.94 eV as RH varies from 10% to 90%. Under similar RH changes, the respective linear ranges for devices with $W_{T}$ of 5 µm, 7 µm, and 9 µm are 0.805–0.92 eV, 0.877–0.916 eV, and 0.742–0.775 eV, respectively.

The $\phi_{b}$ of the graphene-trenched silicon Schottky junction should show an inverse relationship with $W_{T}$. At a reference RH of 10%, $\phi_{b}$ decreases from 0.895 to 0.742 eV as the trench width increases from 3 µm to 9 µm. With a similar increase in trench width and RH of 90%, $\phi_{b}$ decreases from 0.94 to 0.775 eV. This decrease in $\phi_{b}$ is associated with an increased carrier injection ratio and improved coupling energy at the graphene–silicon contact in devices with larger trench widths. This is further facilitated by the slightly higher momentum imparted to carriers for crossing the junction in trenched devices, where the combination of lateral and vertical electric fields helps carriers transport across the junction more easily compared to those moving under the influence of vertical fields alone. As the $W_{T}$ of the device increases, the lateral component of the electric field is enhanced.

The inverse correlation between $\phi_{b}$ and $W_{T}$ for the devices characterized with $W_{T}$ values of 5 µm and 7 µm does not align with the previous explanation. This discrepancy might result from inhomogeneities during the preparation of the semiconducting substrate, the formation of trenches, traps encountered during charge transport, inefficient Ohmic contact between graphene and metallic electrodes, and the quality of the back contact.

The ideality factor is a measure of the quality of the graphene–silicon Schottky interface and typically has values ≥1. An ideality factor of 1 indicates a perfect, theoretically ideal Schottky junction. It is primarily influenced by the semiconductor surface morphology, which is directly affected by trench formations. Secondly, it is intrinsically determined by the quality of CVD graphene growth and the chemical/mechanical processes involved in its preparation before being transferred onto silicon. Any surface contamination can degrade surface homogeneity. When SiO_2_ is etched using BOE, it results in a micro–nano-sized mountainous surface rather than a polished edge. Thirdly, the quality of the interface that CVD graphene forms with silicon at contacting regions significantly affects the ideality factor value.

We plot η of the graphene-trenched silicon Schottky devices with trench widths of 3 µm, 5 µm, 7 µm, and 9 µm in Figure 8b against RH values of 10%, 20%, 30%, 40%, 50%, 60%, 70%, 80%, and 90%. A clear linear decrease in η is observed with increasing RH. Furthermore, η should be directly correlated with $W_{T}$. At an RH of 10%, η increases from 2.15 to 5.4 with an increase in $W_{T}$ from 3 µm to 9 µm. Similarly, with a corresponding increase in $W_{T}$ at an RH of 90%, η rises from 1.68 to 4. This increase is primarily due to the reduced quality of the graphene–silicon interface at larger trench widths. Additionally, as trench width increases, there is a higher likelihood of broken graphene crystals in suspended graphene. These breaks can allow for environmental factors, such as dust, particles, and vapors, to infiltrate the trenches, compromising the interface quality and potentially acting as a permanent source of passivation. This compromises the sensing capability of the device.

From the η–RH curves, for a trench width of 3 µm, η decreases from 2.15 to 1.68 as RH changes from 10% to 90%. For devices with trench widths of 5 µm, 7 µm, and 9 µm, the respective reductions in η are from 4.9 to 2.9, 3 to 2.62, and 5.4 to 4. This linear decrease in η with increasing RH is attributed to the potential filling or elimination of surface defects by water vapor, thereby enhancing the quality of the interface. The direct correlation between η and $W_{T}$ for the devices characterized with trench widths of 5 µm and 7 µm bears the same inconsistency, originating from inhomogeneities experienced during material preparation, device fabrication techniques, and contact formation.

In a graphene–silicon Schottky junction, the $R_{s}$ plays a critical role in overall performance, especially at higher current levels. It includes the bulk resistance of the silicon, resistance at the graphene–silicon interface, and interconnect resistance in the circuitry. The $R_{s}$ affects the forward voltage drop across the junction and the diode’s overall efficiency. Mathematically, the total forward biased voltage (V) of a graphene–silicon Schottky junction can be expressed as follows:

(3) $$V=V_{D}+I_{f}R_{s}$$

where $V_{D}$ is the ideal diode voltage drop depending on $\phi_{b}$ and thermal generation, and $I_{f}$ is the forward current. To minimize power loss and ensure efficient operation, $R_{s}$ should be kept as low as possible. Introducing trenches of varying widths reduces the effective cross-sectional area through which carriers can transport across the interface, leading to an increase in $R_{s}$. The $R_{s}$ increases linearly with $A_{HG}$ and is inversely related to $A_{G-Si}$.

At an RH of 10%, $R_{s}$ increases from 17 kΩ to 102 kΩ with an increase in $W_{T}$ from 3 µm to 9 µm, as depicted in Figure 8c. Similarly, with a corresponding increase in $W_{T}$ and at an RH of 90%, $R_{s}$ rises from 5 kΩ to 66 kΩ. This increase primarily stems from the reduced quality of the graphene–silicon interface at larger trench widths, which effectively reduces $A_{G-Si}$ for carrier transport. Additionally, as $W_{T}$ increases, there is a higher likelihood of broken crystals in the conformally covered graphene sheet.

From all the $R_{s}$–RH curves presented in Figure 8c, for a trench width of 3 µm, $R_{s}$ decreases from 17 kΩ to 5 kΩ as RH changes from 10% to 90%. For other graphene-trenched silicon devices characterized with trench widths of 5 µm, 7 µm, and 9 µm, the respective reductions in $R_{s}$ are from 24 kΩ to 13 kΩ, 47 kΩ to 18.5 kΩ, and 102 kΩ to 66 kΩ. This linear decrease in $R_{s}$ with increasing RH is attributed to the creation of alternative conductive paths through water adsorption and the induction of capacitive effects at the interface, thereby functionally reducing the impedance characteristics of the junction.

The RH is defined as the ratio of existing water vapor in the air to the maximum water vapor that air could hold at a given temperature, typically 25 °C. Using the current at low RH $\left(I_{0.1}\right)$ as a baseline, the $S_{T}$ of the graphene-trenched silicon Schottky junction in vertical charge transport for an RH setting of x is expressed as follows:

$$S_{T}=\frac{I_{x}-I_{0.1}}{I_{0.1}\times ({RH}_{x}-{RH}_{0.1})}\times 100\%$$

where ${RH}_{x}$ ranges from 10% to 80%. $S_{T}$ values are directly related to $A_{G-Si}$, highlighting the number of available states of graphene that can be modified by water vapor adsorption. The $S_{T}$ vs. RH data for devices characterized with $W_{T}$ values of 3 µm, 5 µm, 7 µm, and 9 µm is presented in Figure 8d. The data shows a linear increase with RH and a decreasing trend with increasing $W_{T}$. For $W_{T}$ = 3 µm, $S_{T}$ increases from 0.48 to 0.98 as RH changes from 10% to 80%. Similarly, for $W_{T}$ values of 5 µm, 7 µm, and 9 µm, $S_{T}$ increases from 0.31 to 0.88, 0.15 to 0.84, and 0.001 to 0.75, respectively, over the same RH variations. This linear increase is due to the increased availability of absorbates modifying graphene in contact with the silicon substrate and their interplay with silicon’s band alignment.

**Figure 9:**
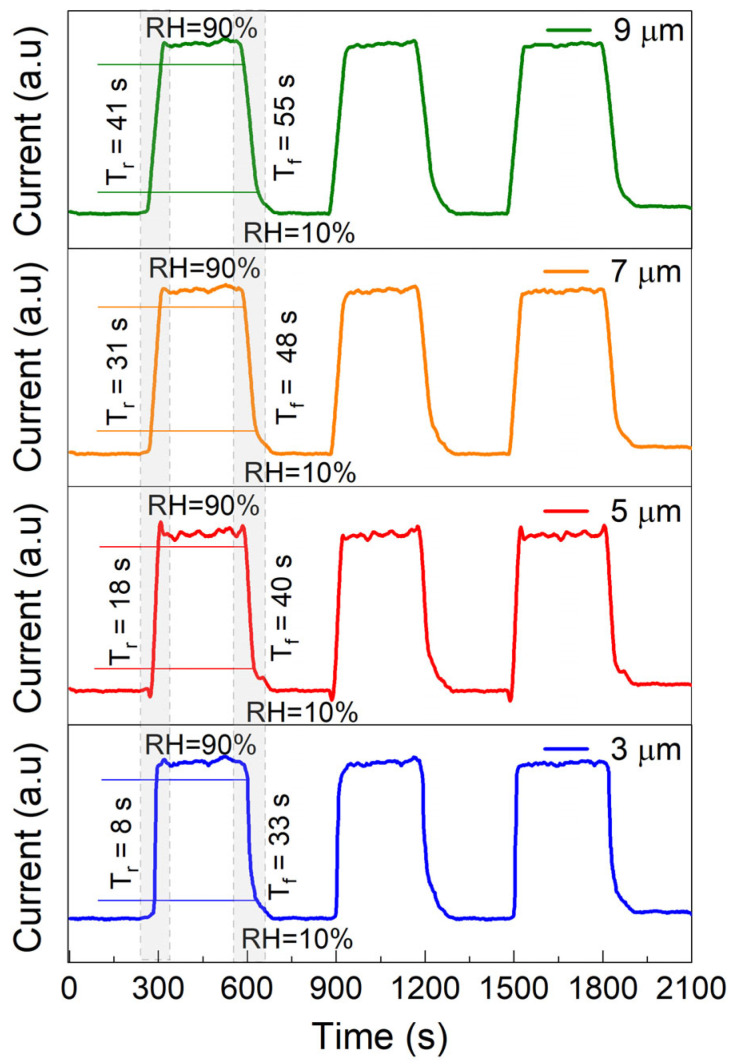
Current–time characteristics of the employed graphene-trenched silicon Schottky junctions characterized with $W_{T}$ of 3, 5, 7, and 9 µm tested under alternating humidity conditions (on/off or high/low switching) are plotted.

Finally, Figure 9 presents the current–time characteristics of the employed devices under alternating humidity conditions (on/off or high/low switching). The rise time $\left(t_{r}\right)$ and fall time $\left(t_{f}\right)$ are defined as the durations required for the current to increase from 10% to 90% and decrease from 90% to 10% of its maximum value, respectively, following a change in humidity level. The response times of the graphene-trenched silicon Schottky junctions are primarily determined by the density of dopable states within the junction region, as suspended graphene is largely insensitive to such environmental variations. The extracted $t_{r}$ and $t_{f}$ values for devices characterized with $W_{T}$ of 3 µm, 5 µm, 7 µm, and 9 µm are 8, 18, 31, and 41 s, and 33, 40, 48, and 55 s, respectively. As $W_{T}$ increases, the density of active states decreases, thereby reducing the device’s sensitivity to humidity-induced changes. The moderate response speed of these devices is partly limited by the humidity control system, particularly the pump/purge cycle time. Additionally, the intrinsic adsorption and desorption kinetics of water molecules on the device surface further contribute to the delayed response. Enhancing the response time could be achieved through improved environmental control systems capable of rapid humidity switching. Prolonged exposure to moisture can impair both the adsorption and desorption processes. Moderate and controlled heating may serve as an effective strategy to maintain desorption efficiency, reduce $t_{f}$, and ensure long-term operational stability.

In the actual atmosphere, besides water vapor, there are other gases such as N_2_ (78.09%), O_2_ (20.95%), Ar (0.93%), and CO_2_ (0.038%). However, these gases minimally affect the humidity sensing capability of the graphene-trenched silicon Schottky diode due to its high selectivity towards water vapor compared to dry gases [55].

For easy comparison of the figures of merit discussed for the graphene-trenched silicon junction devices, Table 1 is provided below.

In this study, we did not consider the possibility of excessive bending where the graphene might bend and make contact with the bottom silicon region in a trench. Additionally, due to the exceptional mechanical strength of graphene, we have disregarded gravitational effects caused by increased RH over suspended graphene. These effects will be examined in our future work, where $W_{T}$ will be increased beyond 9 µm. Our novel heterostructure provides additional sensing capabilities, including light detection within the silicon absorption spectrum and temperature sensing. In future research, we plan to explore the device’s potential in a MESFET configuration, utilizing both freely suspended graphene and graphene in physical contact with silicon as part of the sensing channel. This investigation aims to determine if suspended graphene, which currently serves as an insensitive part of the graphene sheet, can regain its sensing capabilities.

Moreover, by integrating with a simple time-count circuit, we can measure the total exposure durations to light and humidity. This feature allows us to develop an environmental monitoring platform, essential for controlling and monitoring conditions in the cultivation of vegetables, fruits, and plants. Furthermore, these Schottky sensors operate efficiently in low-power mode, which is beneficial for energy conservation and creating environmentally friendly solutions by reducing reliance on electric power cables. This capability is particularly advantageous for monitoring outdoor environments and in large-scale applications, such as e-agriculture fields spanning thousands of acres.

## 5. Conclusions

Sensors employing freely suspended two-dimensional materials present notable fabrication challenges. We report a structurally engineered graphene-trenched silicon Schottky junction that advances humidity sensing by combining suspended and contacted graphene regions within a single device architecture. SEM imaging and Raman spectroscopy confirmed the successful formation of the graphene–silicon junction, while Supplementary Data further verified trench dimensions and surface quality (Figures S2–S5). These structural characterizations directly support the electrical measurements and performance analysis. While suspended graphene is relatively insensitive, it plays a crucial role in regulating the available states for water adsorption on the conformally covered graphene sheet.

The device exhibits a systematic modulation of Schottky parameters as a function of trench width, directly linking junction geometry to sensing performance. The tunable electrical parameters, such as $\phi_{b}$ and $S_{T}$, display an approximately direct linear relationship with RH and an inverse linear correlation with the $W_{T}$. In contrast, the η and $R_{s}$ exhibit an inverse linear relationship with RH and a direct linear correlation with $W_{T}$. Deviations from these trends are ascribed to inhomogeneities during the preparation of the semiconducting substrate, trench formation, charge transport traps, inefficient Ohmic contact between graphene and metallic electrodes, and variations in the quality of the back contact. This multifunctional structure, exhibiting high sensitivity, structural reproducibility, and low-power operation, defines a new paradigm for humidity sensing via suspended 2D interfaces, with direct applicability to industrial safety, environmental monitoring, process control, and smart home systems.

## Figures and Tables

**Table 1:** A comparison of the tunable electrical figures of merit derived from the graphene-trenched silicon junctions with varying trench widths.

Devices	$W_{T}(µm)$	$\phi_{b}(eV)$	η	$R_{s}(kΩ)$	$S_{T}$
**Graphene-Trenched Silicon Junctions**	3	0.895 @ RH_10_ 0.94 @ RH_90_	2.15 @ RH_10_ 1.68 @ RH_90_	17 @ RH_10_ 5 @ RH_90_	0.48 @ RH_10_ 0.98 @ RH_80_
	5	0.805 @ RH_10_ 0.92 @ RH_90_	4.9 @ RH_10_ 2.9 @ RH_90_	24 @ RH_10_ 13 @ RH_90_	0.31 @ RH_10_ 0.88 @ RH_80_
	7	0.877 @ RH_10_ 0.916 @ RH_90_	3 @ RH_10_ 2.62 @ RH_90_	47 @ RH_10_ 18.5 @ RH_90_	0.15 @ RH_10_ 0.84 @ RH_80_
	9	0.742 @ RH_10_ 0.775 @ RH_90_	5.4 @ RH_10_ 4 @ RH_90_	102 @ RH_10_ 66 @ RH_90_	0.001 @ RH_10_ 0.75 @ RH_80_

## Data Availability

Data are contained within the article and Supplementary Materials.

## Supplementary Materials

The following supporting information can be downloaded at: https://www.mdpi.com/article/10.3390/nano15130985/s1, Figure S1: A compact testing kit for humidity and gas characterization, featuring a chamber that accommodates a 2 cm × 2 cm silicon chip with multiple devices. The device selector enables targeted measurements without disrupting the environment, allowing simultaneous data collection from all sensors on the chip; Figure S2: Depth profile of a 3 µm trenched device, showing both the trench depth and width; Figure S3: Depth profile of a 5 µm trenched device, showing both the trench depth and width; Figure S4: Depth profile of a 7 µm trenched device, showing both the trench depth and width; Figure S5: Depth profile of a 9 µm trenched device, showing both the trench depth and width.
