# Supplementary material for: Humidity Sensing in Graphene-Trenched Silicon Junctions via Schottky Barrier Modulation
Source: Nanomaterials (Basel). 2025 Jun 25;15(13):985. doi: 10.3390/nano15130985 (PMC12251016; doi:10.3390/nano15130985)
Supplement: Supplementary file 1 [file nanomaterials-15-00985-s001.zip › nanomaterials-3686194-supplementary.pdf]

Humidity Sensing in Graphene-Trenched Silicon Junctions via Schottky Barrier Modulation

Akeel Qadir [1,2,3], Munir Ali [5], Afshan Khaliq [6], Shahid Karim [1,2], Umar Farooq [7], Hong Sheng Xu [8] and Yiting Yu [2,3,4*]

1 School of Information Engineering, Xi'an Eurasia University, 710065, China.
2 Research Center of Smart Sensing Chips, Ningbo Institute of Northwestern Polytechnical University, Ningbo, Zhejiang 315103, China.
3 Key Laboratory of Micro/Nano Systems for Aerospace (Ministry of Education), Shaanxi, Province Key Laboratory of Micro and Nano Electro-Mechanical Systems, Department, of Microsystems Engineering, Northwestern Polytechnical University, Xi'an 710072, China.
4 Key Laboratory of Scale Manufacturing Technologies for High-Performance MEMS Chips of Zhejiang Province, Key Laboratory of Optical Microsystems and Application Technologies of Ningbo City, Ningbo Institute of Northwestern Polytechnical University, 218 Qingyi Road, Ningbo, 315103 China.
5 Laboratory of Single-Photon Detection and Imaging Techniques, Zhejiang Engineering Research Center for Edge Intelligence Technology and Equipment, School of Information and Electrical Engineering, Zhejiang University City College, Hangzhou, Zhejiang 310015, China.
6 College of Physics and Electronic Information Engineering, Zhejiang Normal University, Jinhua, 321004, China.
7 Department of Mechanical Engineering, University of Colorado Boulder, Boulder, Colorado, 80309, USA.
8 Industry-Education-Research Institute of Advanced Materials and Technology for Integrated Circuits, Anhui 11 University, Hefei, Anhui 230601, China.
* Correspondence: to whom correspondence should be addressed: yyt@nwpu.edu.cn

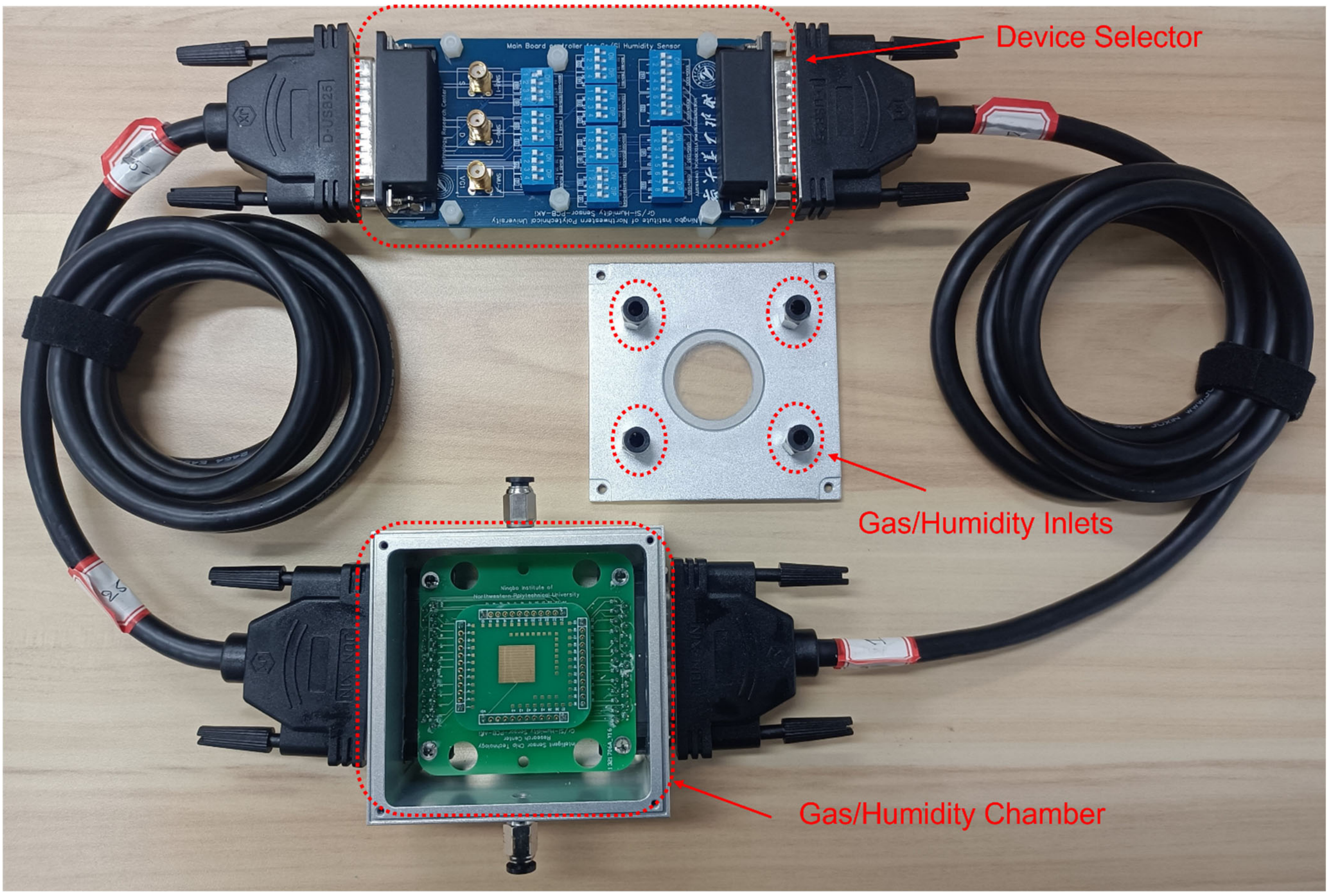

Figure S1. A compact testing kit for humidity and gas characterization, featuring a chamber that accommodates a 2 cm × 2 cm silicon chip with multiple devices. The device selector enables targeted measurements without disrupting the environment, allowing simultaneous data collection from all sensors on the chip.

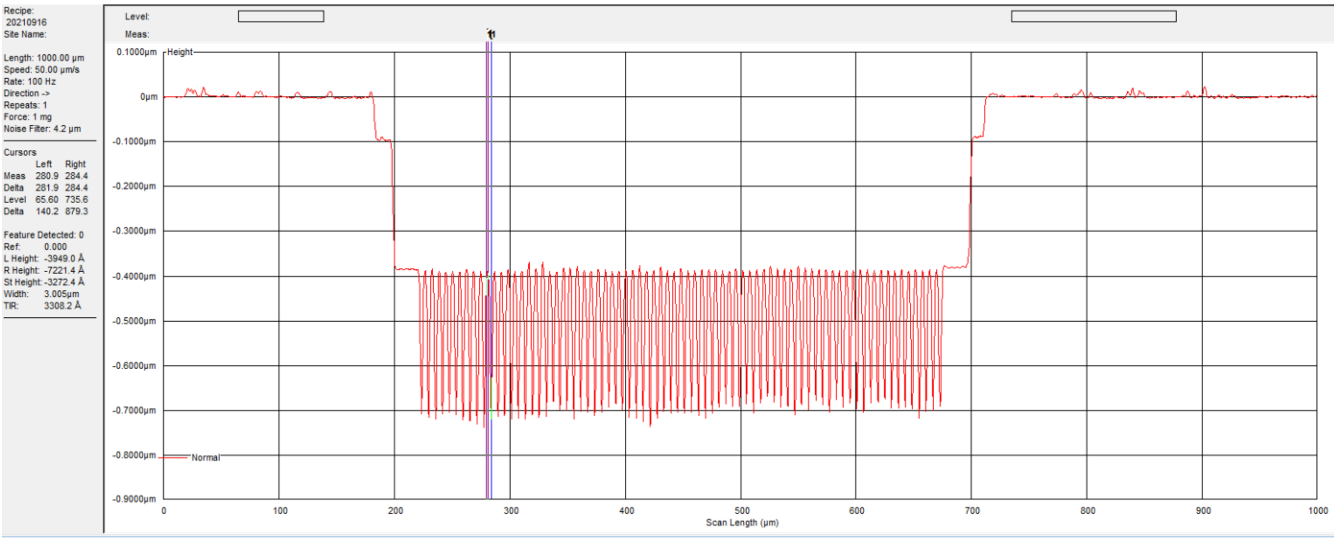

Figure S2. Depth profile of a 3 µm trenched device, showing both the trench depth and width.

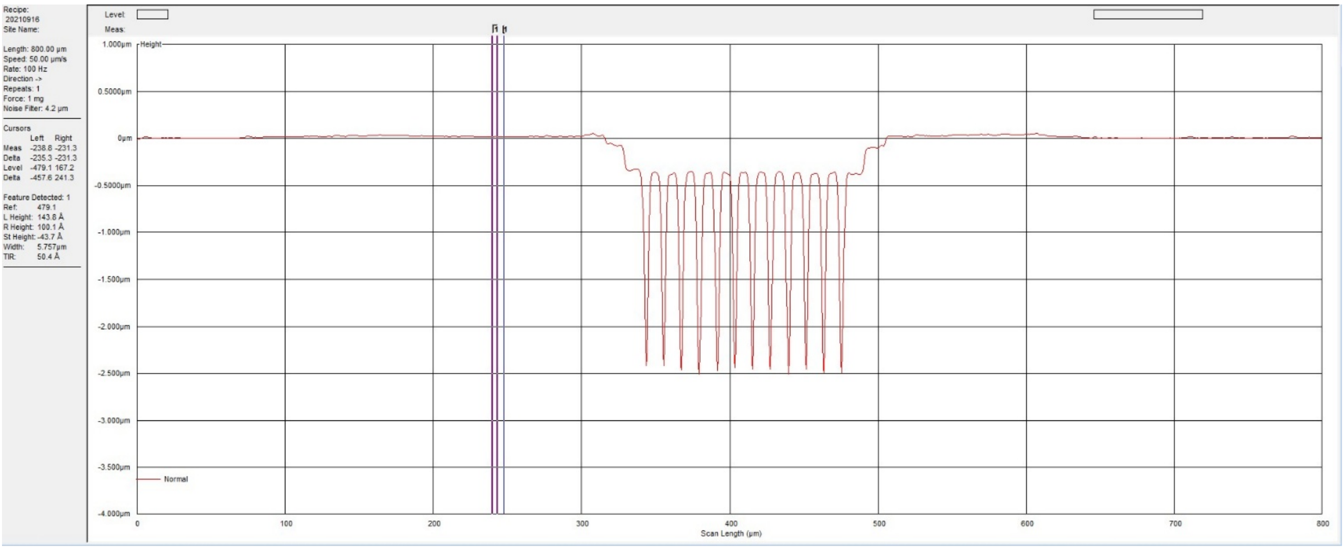

Figure S3. Depth profile of a 5 µm trenched device, showing both the trench depth and width.

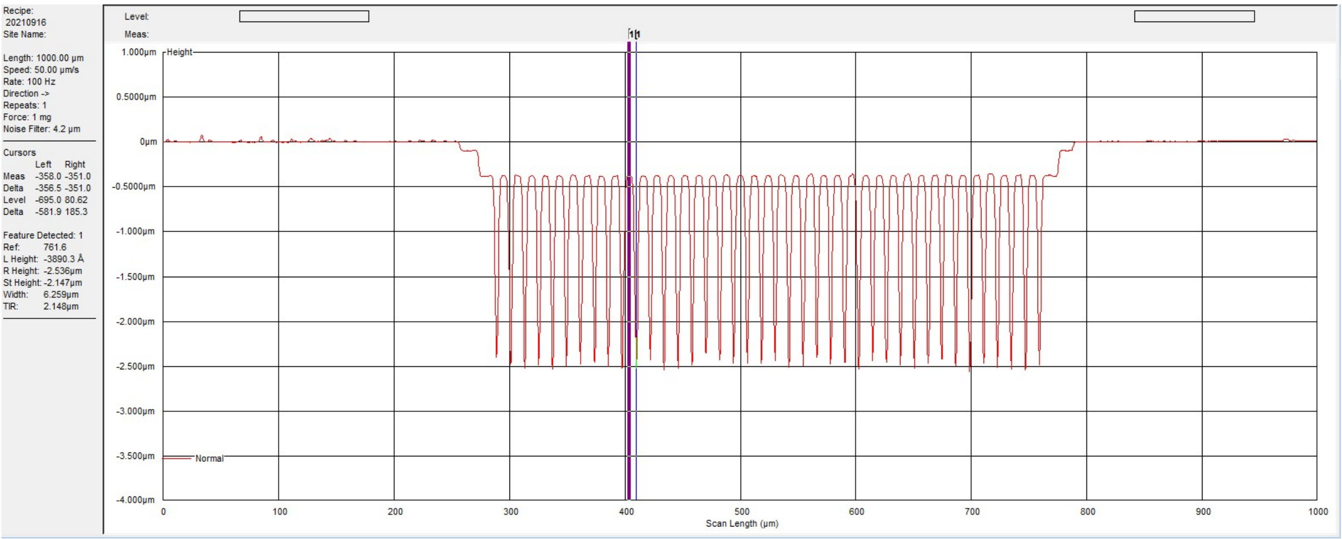

Figure S4. Depth profile of a 7 µm trenched device, showing both the trench depth and width.

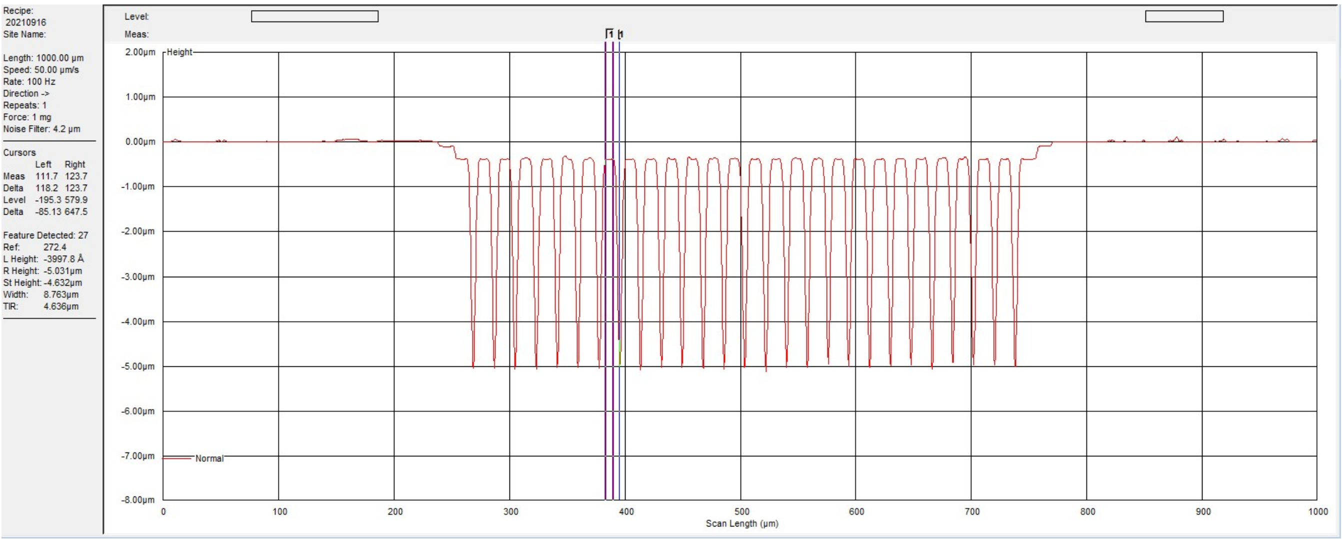

Figure S5. Depth profile of a 9 µm trenched device, showing both the trench depth and width.
